# Sand mining deteriorates soil fertility and farming livelihoods around Accra, Ghana

**DOI:** 10.1038/s41598-024-66656-z

**Published:** 2024-07-24

**Authors:** Katharina Salomea Hemmler, Kofi Yeboah Asare, Emmanuel Yamoah Tenkorang, Andreas Buerkert

**Affiliations:** 1 Organic Plant Production and Agroecosystems Research in the Tropics and Subtropics, Universität Kassel, Steinstrasse 19, 37213 Witzenhausen, Germany; 2 School for Development Studies, University of Cape Coast, Cape Coast, Ghana

**Keywords:** Ecology, Environmental sciences, Environmental social sciences

## Abstract

Urbanization in Sub-Saharan Africa requires vast quantities of sand to meet infrastructural demands. In Accra, Ghana, sand mining occurs predominantly on farmlands at the city’s periphery. We selected three study communities to assess the effects of sand mining on agriculture using interviews with local farmers and soil analyses of mined and unmined fields. The results underscore the profound repercussions of sand mining on farmers, characterized by substantial agricultural land loss, the destruction of food crops, and the absence of compensation payments or land reclamation. The study further reveals a significant decline in soil fertility of mined fields compared to unmined fields as evidenced by reduced levels of carbon (C, by 6.3 g kg^-1^) and macronutrients (nitrogen (N, by 0.6 g kg^-1^), phosphorus (P, 12.7 mg kg^-1^), potassium (K, 77 mg kg^-1^) and magnesium (Mg, 88 mg kg^-1^)), and an increase in soil compaction (bulk density by 0.13 g cm^-3^ and penetration resistance by 0.11 MPa). Soil texture and pH were altered and sodium (Na, by 16 mg kg^-1^) and soil moisture (by 4%) increased. On a communal level, sand mining adversely affected water availability, road infrastructure, and the health of residents. The study fills research gaps on the effects of sand mining on agricultural productivity, soil fertility and livelihoods, emphasizing the urgent need for effective regulation, law enforcement and collaboration as well as compensation and land reclamation mechanisms to limit the adverse impacts of sand mining on ecosystem services. Further, the use of more sustainable building materials should be fostered to reduce the demand for sand in Ghana.

## Introduction

Sand, the main component of concrete, constitutes the skeleton of every city in our modern civilization. It has been conceptualized as the “currency of development”^1^. However, sand mining may also strongly hamper vital ecosystem services and directly or indirectly affect all 17 Sustainable Development Goals (SDGs)^2^. Even though sand is a non-renewable resource in human timescales, it is mined globally at exorbitant scales from rivers, marine environments, and terrestrial deposits, constituting the second most consumed natural resource after water^3^. While global research focusses on open pit and river sand mining in Asia and the Global North, data on terrestrial sand mining in Sub-Saharan Africa (SSA) are limited.

Throughout SSA, cities emerge at an unprecedented speed, making it the world’s fastest urbanizing region^4^. With the urban population of SSA being around 33 million (15% of the total population) in 1960, it reached almost 500 million in 2021, constituting 42% of the region’s total population^5^. SSA’s ever increasing urbanization rate has been driven by rapid inner-urban population growth and rural–urban migration, mainly attributed to the urban–rural gap of job opportunities, infrastructure, education, and health services^6^. At the same time, traditional, more sustainable, building materials such as clay-bricks, bamboo, and straw, are perceived as outdated. As a result, the demand for construction aggregates such as sand, gravel, and laterite, has grown far beyond its natural replenishing rate through weathering and natural erosion or deposition.

The West African country of Ghana is exemplary for SSA’s urbanization pattern. It was the world’s fastest growing economy in 2019 and experiences a continuous economic growth, particularly in the industry and service sector^7^. While agriculture contributes only one fifth of Ghana’s Gross Domestic Product (GDP), it still employs almost 40% of its population^8^. Farming is primarily based on smallholder activities and follows traditional, non-mechanized methods in low input—low output systems^8^. During the last few decades, Ghana’s agricultural land cover (LC) increased considerably but reduced since 2015 with only a slight average increase of 0.7% between 1995 and 2019. During the same time, the LC of built-up areas increased by 132%. The highest expansion of occupied land area occurred in the region of Greater Accra (387 km^2^), followed by the Ashanti, Central, and Western Regions^9^. Consequently, Ghana’s urban population increased by 40% from 2010 to 2021 as compared to 10% of the rural population, with the Greater Accra Metropolitan Area (GAMA) being the largest and fastest growing region providing home to 5 million inhabitants^10^. The urban sprawl of Ghana’s political, economic, and cultural capital leads to a depletion of natural resources in the peri-urban fringes, showcasing the link between urbanization and sand mining. To meet the infrastructure needs, daily estimated 765 truckloads of sand are mined from the hinterlands and transported to the city, leading to a yearly volume of 4.55 million m^3^ of sand^11^. The desired mineral is sourced from farmlands in peri-urban and rural areas, extending up to 60 km from the city centre. The mining, locally also referred to by the euphemism of “sand winning”, is organized by sand miners who use heavy machinery to remove sand from the earth’s surface. The process begins with the clearing of land, including vegetation, sometimes food crops or even tree plantations, and the topsoil. Afterwards, payloaders are used to place the sand onto tipper trucks which then deliver it to the customer, mostly individuals and block manufacturers^11,12^. Typically, the mined land is left without reclamation, meaning that it is not restored to its natural state, but rather revisited by sand miners after some months or years of replenishing through the erosion of neighbouring fields (the so called “gari effect”)^11^. Governmental regulations to control sand mining and to foster post-mining reclamation are in place but ineffective, due to a combination of bureaucracy, corruption, and insufficient law-enforcement. While sand mining is very profitable for a small number of people (sand miners, landowners, and tipper truck owners) and necessary for urban development, it has far-reaching negative consequences for the social-ecological system it is part of. Costs are externalized onto the environment and the local population of sand mining communities, mainly farming families. Since Accra also depends on the surrounding rural areas for food production, sand mining and agriculture are rivals in the competition for land.

In view of the above, this paper aims at closing research gaps by (i) analysing the effects of sand mining on agricultural productivity, (ii) assessing the effect of sand mining on soil fertility through a comparison of physical and chemical parameters of mined and unmined fields, and (iii) determining effects of sand mining on the livelihoods of mining communities.

## Materials and methods

### Study area

Located in the South of Ghana, Accra (5°32′48.26″N, 0°12′34.33″W, 20 m above sea level (a.s.l)) lays in the agro-ecological zone of the Coastal Savannah^13^. It experiences an annual rainfall of 757 mm (10 year average from 2012 to 2021)^8^, an average temperature of 25.1 °C during the coldest month (August) and of 28.4 °C in the warmest month (March/February)^14^. Its tropical climate is characterized by a bimodal precipitation distribution with rainy seasons from April to July and from September to October. These define the major and minor farming seasons. Multi-cropping and mixed cropping are common practices, with a focus on smallholder farming^15^. The most cultivated crops in the Greater Accra Region are staples like maize (*Zea mays* L.), and cassava (*Manihot esculenta* Crantz), as well as vegetables including pepper (*Capsicum annuum* L. *var. annuum*), okra (*Abelmoschus esculentus*), Ethiopian nightshade (*Solanum aethiopicum* L.), cabbage (*Brassica oleracea* L. Capitata group), and tomato (*Lycopersicon esculentum* Mill.) as well as fruits such as pineapple (*Ananas comosus* (L.) Merr.), watermelon (*Citrullus lanatus*), pawpaw (*Carica papaya* L.), and mango (*Mangifera indica* L.)^14^.

**Figure 1:**
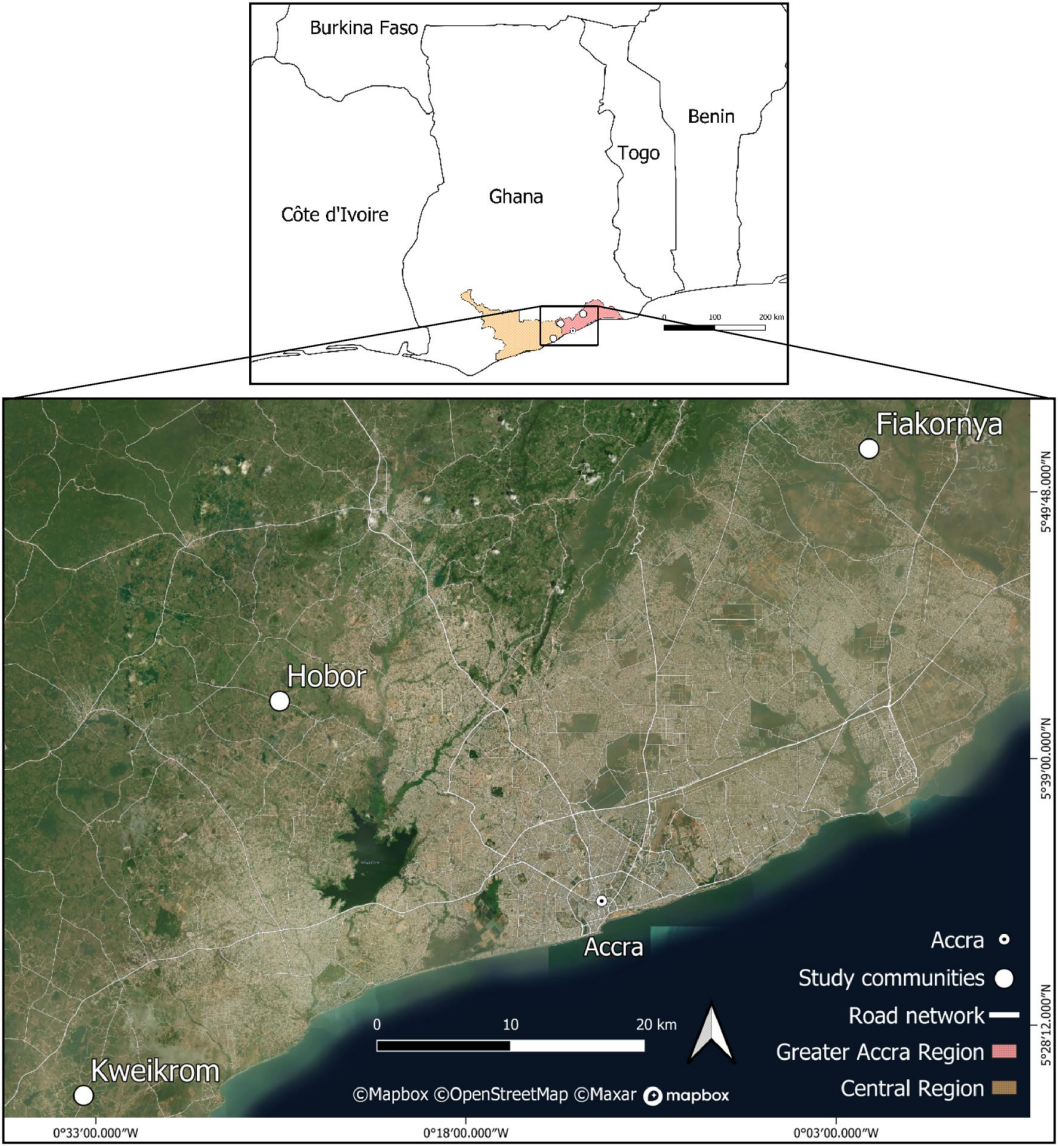
Location of the study areas in southern Ghana, West Africa. The figure was generated using QGIS (Quantum Geographic Information System, version 3.16, http://qgis.osgeo.org), Mapbox (https://www.mpbox.com/), and OpenStreetMap, available under the Open Database License (https://www.openstreetmap.org/copyright).

The communities of our study were selected purposively based on two criteria: First, agriculture is the mainstay for most of the population with both subsistence and cash-crop farming being practiced. Second, sand mining has been prevalent in these communities for several years and is still ongoing at a considerable rate. Therefore, the communities of Hobor (Greater Accra Region, Ga South district, 5°41′20.1″N, 0°25′33.8″W, 50 m a.s.l.), Kweikrom (Central Region, Gomoa East district, 5°25′19.9″N, 0°33′29.3″W, 55 m a.s.l) and Fiakornya (Greater Accra Region, Shai Osudoku district, 5°51′32.8″N, 0°01′40.1″W, 45 m a.s.l) were chosen (Fig. 1). They are situated at the fringe of Accra, with beeline distances to the city centre of around 40 km. Hobor is a migrant community with most of its estimated 2600 inhabitants being *Ewe* settlers. While they cultivate the fields, the lands are mostly possessed by a few families of the *Ga* tribe. Kweikrom is an indigenous *Fante* community with an estimated population of 800 people. The chief, constituting the traditional, cultural leader of the community, and his family claimed to own all the lands, but this has been contested by some other family heads at the court. Fiakornya is populated by an estimated 1000 Ga*-Adangbe* people and lands are possessed by several families.

### Data collection and analysis

To assess the spatial dimensions and development of mining activities, we aimed to undertake time series remote sensing analyses. After an initial manual classification of sand mining sites within a 100-km radius around Accra to identify potential mining sites, we further intended to carry out a supervised classification of mining areas using the open-source software QGIS(Quantum Geographic Information System ,version 3.16, http://qgis.osgeo.org). However, several constraints hindered the continuation of this endeavour: high cloud-coverage during the rainy season, limited availability of high-resolution satellite imagery, high level of misclassification such as of unpaved roads and communities as mining sites, and the dynamic nature of mining activities (landscape reconstitution, repeated mining at the same site, gradual expansion of mining sites on a daily basis).

Primary data collected for the study were obtained from structured and unstructured interviews as well as soil analyses. A total of 98 interviews were conducted with farmers in Hobor (n = 35), Kweikrom (n = 27) and Fiakornya (n = 36) between April and August 2021. Within each location, farmers were selected using a simple random sampling technique while for data collection, the opensource software Census and Survey Processing System (CSPro, version 7.5, U.S. Census Bureau, 2020, https://www.census.gov/data/software/cspro.html) was employed. The interviews were translated from English to local languages, including Twi, Ewe, and Ga. In accordance with their guidelines and regulations, the ethical approval by the central ethics committee of the University of Kassel was obtained. Informed consent was obtained from all participants. The semi-structured questionnaire contained four parts: i) socio-demographic data, ii) sand mining on agricultural land (number and size of mined fields, previous cultivation practices, post-mining land use), iii) current agricultural activity (crops, quantities of inputs and outputs, cost-revenue structure), and iv) effects of sand mining on the community and environment. It needs to be noted that the second and third part did not apply to all farmers, because some had not experienced sand mining on their land and others completely stopped farming after sand had been mined on their fields. Unstructured interviews were conducted with different sand mining stakeholders, including landowners (private and real estate companies), sand miners, truck drivers, block manufacturers, and regulating agencies (Minerals Commission, Environmental Protection Agency, and local government authorities).

Soil data were obtained from mined land (previous agricultural field) and cultivated agricultural fields, one adjacent to the mining site and one further away. This set of three fields was repeated nine times in Hobor and Fiakornya and ten times in Kweikrom, leading to 28 sets (84 fields). For each field, total area and corner coordinates were recorded using the GPS device eTrex 20 (Garmin International Inc., Olathe, KS, USA) and a division into upper and lower slope was made to account for erosion effects. For every field, an analysis of chemical and physical properties was undertaken: Soil moisture was measured 18 times per field (nine times upper and lower slope each) using the soil moisture meter Fieldscout TDR350 with 7.6 cm rods from the soil surface (Spectrum Technologies Inc., Aurora, IL, USA). The total number of measurements had to be reduced from 1512 to 1379 because several mined fields were too rocky/compacted to allow measurements. For bulk density, a steel ring (height = 3.9 cm, volume = 112 cm^3^) was used to collect six undisturbed samples per field (three upper slope, three lower slope, total amount reduced from 504 to 470). All samples were dried at 105 °C for eight hours and weighed. Soil compaction was measured at 0–10 cm depth with a pocket penetrometer (Royal Eijkelkamp B.V., Giesbeek, The Netherlands) at six points per field, with ten replications at each point. For chemical soil analysis, 18 sub-samples per field were collected at 0–10 cm depth and pooled into six samples (three upper slope, three lower slope). As described by Motsara and Roy^16^ all samples were sieved to < 2 mm particle size and air-dried prior to analyses of soil pH, carbon/nitrogen (C/N) ratio, and plant available nutrients (phosphorus (P), potassium (K), calcium (Ca), magnesium (Mg), and sodium (Na)). For soil pH, 5 g of soil were diluted with 12.5 ml of demineralized water and measured with a pH meter (WTW ProfiLine pH 3110, Xylem Analytics Germany Sales GmbH & Co. KG, Weilheim, Germany). Soil total C and N were determined using a thermal conductivity detector (Vario MAX CHN Analyser, Elementar Analysensysteme GmbH, Hanau, Germany). A selective measurement of carbonate content from pooled fields using the Scheibler method revealed that carbonates were < 2.5%. Therefore, total C-values correspond to organic C. Plant available nutrients (P, K, Ca, Mg, and Na) were assessed with an Inductively Coupled Plasma—Optical Emission Spectrometry (Spectrogreen FMX46 / 2020, SPECTRO Analytical Instruments GmbH, Kleve, Germany) after a calcium-acetate-lactate (CAL) extraction (2.5 g of soil with 50 ml of CAL extraction solution)^17^. After pooling the subsamples per field, soil particle size was analysed for nine fields per community^18^ to obtain contents of sand, silt, and clay.

Descriptive statistics, t-tests, ANOVA, Pearson’s Chi-square test were performed using R 4.1.2 software^19^, RStudio^20^, and the Tidyverse package^21^. To assess the potential effects of sand mining and erosion on soil chemical and physical properties, a Liner Mixed Model (LMM) was fitted. Tukey’s Ladder of Powers was used for normal distribution and the nested ANOVA designated mining status and slope (and their interaction) as fixed effects and community and field number as random effects to account for potential variability between communities and individual fields, thus improving the robustness of the analysis (nlme package)^22^. Furthermore, the Compact Letter Display (CLD) of the multcomp package was employed to determine significant differences between unmined, near mined and mined fields as well as lower and upper slope areas.

## Results

### Socio-economic and agricultural characteristics

The 98 interviewees (47% women, 53% men) were on average 51 years old (24 to 75 years). On average, they had a formal education of five years, whereby the mean for women (3.5 years) and elderly people (70–80 years: 0.9 years) was considerably lower than for men (6.3 years) and young interviewees (20–30 years: 11.8 years; t-test for gender: *P* = 0.008; ANOVA for 10-year interval of age group: *P* = 0.009). Formal education was higher in Hobor (average of 6.8 years) than in Fiakornya (3.9 years) and Kweikrom (4.0 years). Average household size was 7.4 family members with no significant differences between age groups or communities (ANOVA for 10-year age interval: *P* = 0.27 and for communities: *P* = 0.76). The average interviewee farmed for 27.2 years irrespective of gender (t-test: *P* = 0.9) and community (ANOVA: *P* = 0.76). Farmers heavily depended on agriculture since 76% stated that it was their main source of income livelihood. Almost half (48%) of the respondents had and outside farm income from small-scale trading (n = 10), sale of charcoal or firewood (n = 10), work in food or agriculture related jobs (n = 7), and construction (n = 6). The obtained off-farm monthly income ranged from 7 US$ to 852 US$ per month with an average of 147 US$ (exchange rate of GHS 1 = 0.17033 US$, Oanda Currency converter, 30.06.2021).

The farmers cultivated on average one ha of land (equalling 1.8 plots with a mean plot size of 0.57 ha). Crops most cultivated were maize (n = 47), cassava (n = 29), pepper (n = 27), and groundnut (*Arachis hypogaea* L., n = 24), followed by pineapple, tomato, and okra. All communities included maize and cassava in their top three crops, whereas the third crop varied (Hobor: pineapple, Kweikrom: groundnut, Fiakornya: pepper). On 19% of the fields, a mixed cropping system was used, with maize-cassava as the most common crop combination. About 35% of the fields were owned by the farmers, whereas 65% belonged to family heads or traditional chiefs. Nevertheless, 53% of the rented fields were given out to the farmers for free, however, major differences were noted between Kweikrom (90%), Fiakornya (42%), and Hobor (4%). Stated reasons were that landowners gave out land to farmers to take care of it (Kweikrom) and when the land was bought by individual developers, farmers were allowed to continue working on it until construction started (all communities). The average annual rent in Hobor was 123 US$ ha^-1^ (21 to 253 US$ ha^-1^) and in Fiakornya 56 US$ ha^-1^ (30 to 140 US$ ha^-1^). Irrigation only played a minor role as 92% of the fields were rainfed. While 60% of the fields were tilled by tractors and 40% by hand, there was a significant inter-communal difference for the rate of mechanization (Hobor and Fiakornya 77% *versus* Kweikrom 34%; Pearson’s Chi-square test: *χ*^2^ = 23.922, *P* = 6.389e^-06^). The average price paid for land tilling was 128 US$ ha^-1^. On 45% of the fields, chemical fertilizer, the most common being NPK, urea, and ammonia, was applied, with no significant difference between the communities (*p* = 0.2). Average costs for fertilization were 133 US$ ha^-1^. On only 9% of the fields, organic fertilization, predominantly as cow manure, was applied and on 48% of the fields, chemical insecticides were used. For weed control, both, manual weeding and herbicides were common (herbicides only: 7%, manual weeding only: 28%, herbicides and manual weeding: 65%). Farmers spent 63 US$ ha^-1^ on herbicides and 290 US$ ha^-1^ for labour to weed and/or apply herbicides. For planting and harvesting, only 24% and 33% of the fields required hired labour, with average costs of 168 US$ ha^-1^ and 231 US$ ha^-1^, respectively. Farmers often used extended family labour, who were provided with meals or agricultural produce for planting or harvesting. On average, 3.8 family members regularly worked on the field(s) at 4.5 days per week. Agricultural products were sold to local markets (all crops) or consumed by household members (mainly maize and cassava, Table 1). About 90% of the farmers noticed yield fluctuations on their fields resulting from weather conditions and response to inputs, while 8.4% reported stable yields, and 1.4% were undecided. All farmers experienced major price fluctuations, depending on market conditions and season (Table 1).

**Table 1:** Consumption, average yield and average selling price of the five main crops grown in the study areas in southern Ghana (2021).

Crop	Household consumption (%)	Sale to market (%)	Yield (t ha ^-1^)	Price (USD t ^-1^)
Maize	60.6	39.4	2.05	216.31
Cassava	46.9	53.1	4.18	337.83
Pepper	5.4	94.6	1.98	564.54
Groundnut	4.7	95.3	1.53	364.60
Pineapple	0.1	99.9	10.31	226.12

### Effects of sand mining on agricultural productivity, farmer’s livelihoods, and future perspectives

**Figure 2:**
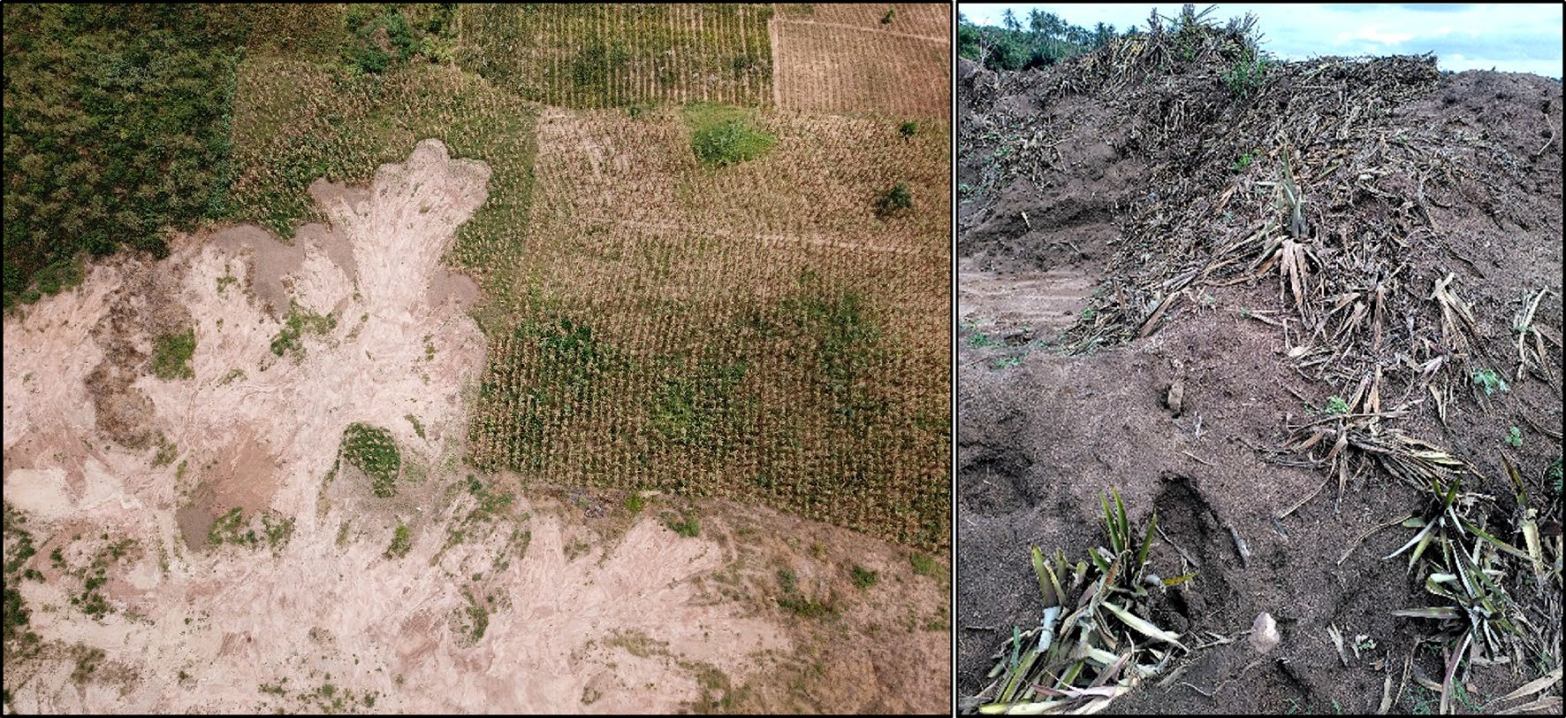
Loss of agricultural harvest (left: maize, right: pineapple) due to sand mining in the study area of southern Ghana (June to August 2021).

In the studied communities, sand mining had occurred year-round for several years. In Kweikrom, mining commenced in the year 2000, in Hobor in 2011, and in Fiakornya in 2013. Thereby, 63% of the interviewed farmers had experienced sand mining on their fields. In total, 121 fields (95.7 ha) were mined. Around 40% of the fields were harvested completely or remained uncultivated at the point of mining so that no yield loss occurred, while 23% experienced some yield losses and 36% a complete loss (Fig. 2). Combining the lost area per crop with the yields of the main crops (Table 1), an annual loss of 106 t pineapples (10.3 ha), 53 t maize (26 ha), 48 t cassava (11.5 ha), 12 t pepper (5.8 ha), and 10 t groundnut (6.3 ha) was calculated.

Sand mining occurred during night-time (36.7%, 34 ha), day-time (32.5%, 23 ha), and during day and night (30.8%, 38 ha). Certain fields were mined incrementally, while most of them completely disappeared within 24 h. Therefore, the farmers stated a ratio of mined size to total field size between 50 and 100%, averaging at 95%. Some sand mining sites were remined after months to years. Over 90% of the farmers were not informed beforehand about the planned mining activity, while around 5% were approached by miners and another 5% were contacted by the landowners prior to land loss. Consequently, there were no compensation payments for most of the farmers (90%) and the remaining 10% received cash compensation totalling between 7 and 51 US$ per plot. Land reclamation was hardly undertaken, only 3.5% of the fields were levelled, 4.3% were partly or sporadically reclaimed, and 92.2% remained without any reclamation once mining was finished.

Farmers reported a loss of soil quality (104 field counts), increased soil compaction (94 counts), water issues, and soil erosion (each 89 counts) on the mined fields. Effects on surrounding fields were minor (water issues (5 counts), soil erosion (9), destruction of farmland by sand mining machinery and workers (8), and dust on plants (6)) as most of the neighbouring fields were equally mined (87 counts). Farmers did not expect the land to recover in the near future, as they estimated a soil regeneration time of > 20 years for 89% of the fields (0–10 years for 10% and 10–20 years for 1% of the fields). Accordingly, on 97% of the fields, the farmers had not restarted agricultural activity and for the few fields where agriculture had restarted, yields were drastically reduced. Three quarters of the mined fields were sold as building plots, 22% remained idle, and on 4%, farmers had no information.

Most farmers had no response strategy to their fields being mined and therefore had to accept reduced harvests and income (59%), while 35% relocated to another field (mostly combined with a reduction in farm size or a switch to backyard gardening) and 6% changed their occupation. One fifth of the farmers had stopped cultivation completely while four fifths of the farmers wanted to continue farming in the future as the vast majority had few (38%) or no (61%) job alternatives. Due to sand mining, most (54%) of the farmers saw no future for agriculture in their community, compared to seeing a future (35%) and being unsure (10%). In contrast to that, 44% of the farmers expected a continuation of sand mining activities, 34% did not, and 22% were unsure. Almost two thirds (64%) of the farmers argued for a complete ban of sand mining through collaborative law enforcement by government agencies, landowners and community elders while 18% preferred improved regulations and 19% were unsure about any effective solution.

### Effects of sand mining on soil chemical and physical parameters

**Figure 3:**
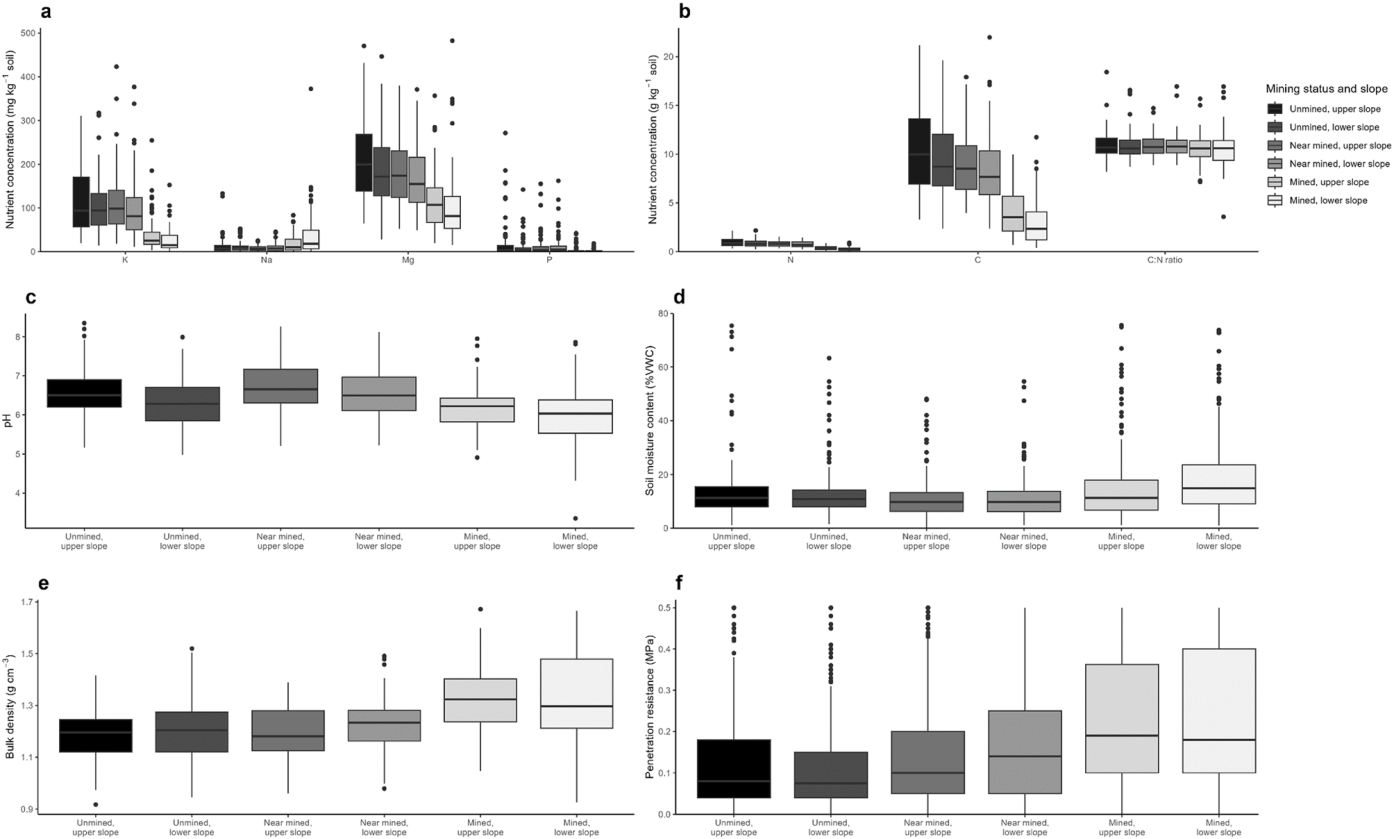
Boxplots of soil chemical and physical properties by mining status and slope at mining locations in southern Ghana (2021). (**a**) Plant available nutrients (potassium (K), sodium (Na), magnesium (Mg), phosphorus (P)). (**b**) Total nitrogen (N), carbon (C) contents and C:N ratio. (**c**) Soil pH levels. (**d**) Soil moisture content. (**e**) Bulk density of the soil. (**f**) Soil penetration resistance. Boxplots show the median, the 25% and 75% quantiles with whiskers marking up to 1.5 of the inter-quartile range, and points indicating outliers.

Sand mining significantly affected all measured soil chemical and physical properties except for the C/N ratio (Table 2 and Fig. 3). It significantly reduced soil contents of available K, Mg, P, N, and C as well as pH, compared with the near mined and unmined sites. Thus, average reductions between unmined and mined fields were noted to amount to 77 mg K kg^-1^, 88 mg Mg kg^-1^, 12.7 mg P kg^-1^, 0.6 g N kg^-1^, 6.3 g C kg^-1^, and 0.32 pH units, while Na increased by 16 mg kg^-1^, soil moisture by 4%, bulk density by 0.13 g cm^-3^, and penetration resistance by 0.11 MPa. Mining changed particle size distribution towards higher sand and lower silt and clay contents (Table 3). Soils at upper slope positions had significantly higher K (+ 12 mg kg^-1^), Mg (+ 15 mg kg^-1^), N (+ 0.1 g kg^-1^), C (+ 0.8 g kg^-1^), and pH (0.23 units) and lower soil moisture (- 1.2%), compared with those at lower slope positions. There was only little effect (mainly for penetration resistance but also soil moisture, P, N, and C) of the interaction of mining status and slope position (Table 2).

**Table 2:** Linear Mixed Model (LMM): F-values and significance levels (*P < 0.05; **P < 0.01; ***P < 0.001) for the fixed effects of mining status and slope (both simple and interaction effects) on soil chemical and physical properties (potassium (K), sodium (Na), magnesium (Mg), phosphorus (P), total nitrogen (N), total carbon (C), C:N ratio, soil pH levels, soil moisture content, bulk density of the soil, and soil penetration resistance) at mining locations around Accra, southern Ghana (2021).

Variable	Mining status	Slope	Mining Status x Slope
K (mg kg^-1^)	45.50***	25.41***	2.77
Na (mg kg^-1^)	12.13***	3.56	2.64
Mg (mg kg^-1^)	18.25***	23.51***	0.43
P (mg kg^-1^)	23.40***	3.33	5.97**
N (g kg^-1^)	58.17***	31.32***	5.16**
C (g kg^-1^)	51.17***	28.94***	4.29*
C/N ratio	1.18	0.06	0.17
pH	8.07***	29.32***	0.47
Soil moisture (%VWC)	4.96**	8.10**	6.91**
Bulk density (g cm^-3^)	44.65***	2.68	0.70
Penetration resistance (MPa)	11.41***	5.85*	9.81***

**Table 3:** Mean values ± standard error of the mean of soil chemical and physical properties (potassium (K), sodium (Na), magnesium (Mg), phosphorus (P), total nitrogen (N), total carbon (C), C:N ratio, soil pH levels, soil moisture content, bulk density of the soil, soil penetration resistance, percentages of sand, silt, and clay) by mining status and slope at mining locations around Accra, southern Ghana (2021). Different letters indicate significant differences for mining status (averaged over all slope levels) and slope (averaged over all mining status levels) at P < 0.05.

	Mining status				Slope	
Variable	Unmined	Near mined	Mined		Lower	Upper
K (mg kg^-1^)	110 ± 5.4 a	104 ± 5.5 a	33 ± 2.7 b		76 ± 4.3 a	89 ± 4.6 b
Na (mg kg^-1^)	12 ± 1.3 a	9 ± 0.6 a	28 ± 3.2 b		19 ± 2.2 a	13 ± 1.1 a
Mg (mg kg^-1^)	203 ± 7.7 a	173 ± 5.8 a	115 ± 6.7 b		156 ± 5.9 a	171 ± 1.2 b
P (mg kg^-1^)	16 ± 2.8 a	12 ± 1.7 a	3 ± 0.5 b		9 ± 6.0 a	12 ± 1.9 b
N (g kg^-1^)	0.9 ± 0.03 a	0.8 ± 0.02 a	0.3 ± 0.02 b		0.65 ± 0.03 a	0.72 ± 0.03 b
C (g kg^-1^)	9.6 ± 0.3 a	8.7 ± 0.3 a	3.5 ± 0.2 b		7.1 ± 0.3 a	7.8 ± 0.3 b
C/N ratio	10.9 ± 0.1 a	10.9 ± 0.1 a	10.6 ± 0.1 a		10.8 ± 0.1 a	10.8 ± 0.1 a
pH	6.4 ± 0.1 a	6.6 ± 0.1 a	6.1 ± 0.1 b		6.3 ± 0.1 a	6.5 ± 0.04 b
Soil moisture (%VWC)	13 ± 0.4 ab	11 ± 0.3 a	17 ± 0.8 b		14 ± 0.4 a	13 ± 0.4 b
Bulk density (g cm^-3^)	1.19 ± 0.01 a	1.22 ± 0.01 a	1.32 ± 0.01 b		1.25 ± 0.01 a	1.23 ± 0.01 a
Penetration re-sistance (MPa)	0.12 ± 0.003 a	0.16 ± 0.003 a	0.23 ± 0.004 b		0.17 ± 0.003 a	0.17 ± 0.003 b
Sand (%)	71 ± 2.0 a	75 ± 1.0 a	80 ± 1.6 b			
Silt (%)	21 ± 9.2 a	11 ± 0.8 a	9 ± 0.9 a			
Clay (%)	15 ± 1.8 a	11 ± 0.8 ab	9 ± 1.1 b			

### Community effects of sand mining

**Figure 4:**
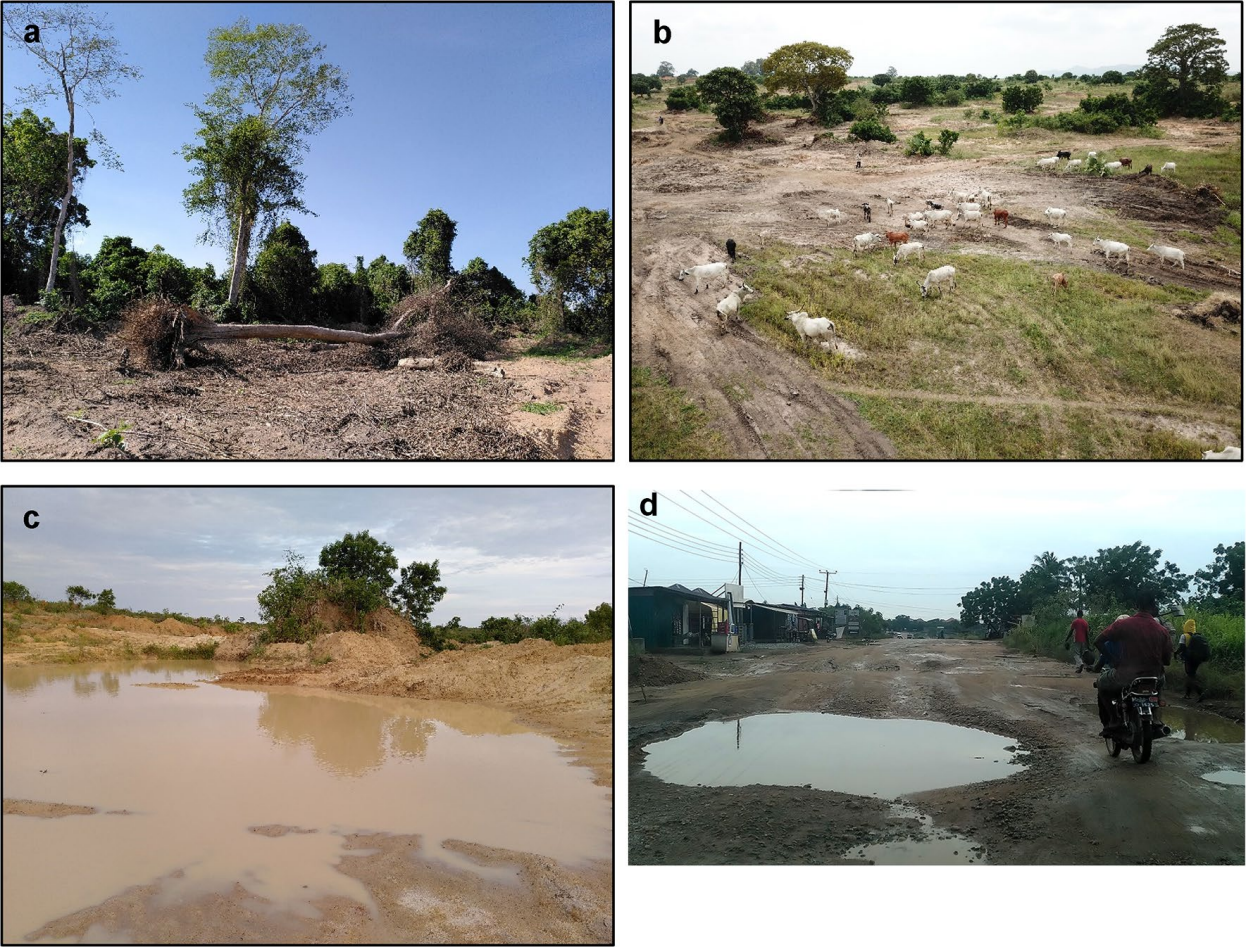
Effects of sand mining around Accra, southern Ghana (June to August 2021). (**a**) Loss of trees due to land clearing for sand extraction. (**b**) Reduction of cattle grazing areas. (**c**) Creation of water ponds becoming mosquito breeding grounds. (**d**) Deterioration of road infrastructure due to frequent passing of heavily loaded sand trucks.

As a consequence of the loss of farmland, farmers recognized the loss of trees and natural vegetation as mining-induced problems with negative effects such as changes in microclimatic conditions leading to a hotter and less aerated climate, loss of firewood, reduced availability of natural medicinal herbs, destruction of wildlife habitat leading to snake encroachment into the communities, and a lack of cattle grazing areas (Fig. 4a,b). The latter also caused conflicts among herders and crop farmers as there were incidents of cattle grazing on farmland.

Furthermore, 87% of the farmers stated that sand mining had negative effects on water conditions (6% no effect, 1% positive effect, 6% no answer or unsure). The most mentioned associated problems were the creation of waterlogged areas (Fig. 4c), the disruption of river flows such as blocking of waterways by silt leading to flooding, the reduction in river water quality through pollution, and the destruction or sand coverage of communal watering holes. The only positive aspect mentioned was the provision of water for domestic use from ponds created by the miners.

Most farmers (73%) reported negative effects on their health, while 13% each perceived no effects or were unsure; none recognized positive effects. The negative effects could be further classified into dust-related health issues (such as asthma and catarrh, n = 25), increased risk of malaria due to water gullies becoming mosquito breeding spots (n = 21), lack of clean water for drinking and hygiene, as well as related infections (n = 16, with 12 counts in Kweikrom), psychological problems due to the loss of farmland or the fear of it (n = 5), nutritional effects (decreased food diversity and quantity, n = 3), and noise pollution through passing trucks, especially at night (n = 2).

Farmers also noted a negative effect of sand mining on the road infrastructure (81%; 10% no effect, 2% positive effect, 6% no answer or unsure). The main reason assigned by the farmers was the destruction of roads by the creation of potholes due to the passage of heavy trucks and mining machinery (Fig. 4d). Moreover, farmers mentioned that it had become difficult to build (finding land, e.g., for community buildings, construction on mined land) and therefore argued that their community was not developing despite the abundance of sand as everything had been sold out. It was further mentioned that infrastructure was damaged due to nearby sand mining activities (flooding of houses, erosion of building foundations).

Other negative effects were the increased prices of food items, higher transportation costs to reach the farms, the relocation of family members (either to rural areas to continue farming or to Accra to find another occupation), increased cleaning work due to dust in the houses, and increased land litigation conflicts within the community.

Those who mentioned positive effects cited the creation of untarred roads to the farming areas through the continuous passage of tipper trucks. Further positive effects were easy access to sand for construction and income for the youth working as support staff such as those who led trucks to sand mining sites. Overall, 24 farmers reported at least one positive effect of sand mining on community livelihoods, while 96 farmers mentioned one or more negative effects.

## Discussion

The farming system of the interviewees was typical for much of southern Ghana, characterized by small farm sizes, strong dependency on rainfall, and reliance on maize and cassava as staples. In contrast to a recent report by Ghana’s Ministry of Food and Agriculture which refers to little mechanized farming and prevalence of intercropping^8^, farmers in our study areas strongly relied on mechanical equipment (tractors), cash crop production, and close market access reflecting their proximity to Accra.

Reductions in farm size and crop yield as a consequence of sand mining were also reported by Anokye et al.^23^ for the Central Region of Ghana. Around Accra, a loss of livelihoods in farming communities due to sand mining was found^11^. Similarly, a recent paper by Asare et al. untangled different notions of sand and (in)security in terms of food, livelihood, water, and health^24^. Further, it was found that 57% of farmers that were affected by sand mining around Accra adopted alternative livelihood strategies which improved food consumption, employment and resilience but were less sustainable compared to farming^25^.

Our findings revealed a significant deterioration in soil chemical and physical properties due to sand mining, except for soil moisture, which slightly increased with sand mining. This phenomenon may reflect an increased soil compaction on the one side and the creation of waterlogged areas on the other side. Due to the compacted or rocky surface soil, little water can be retained in the topographically upper laying parts of the mined fields, which is therefore running off and collecting in the lower slope areas. Saviour and Stalin^26^ investigated physico-chemical soil parameters of sand mining sites in Tamil Nadu, India. They observed that the water infiltration rate of mined, rehabilitated fields increased compared to unmined land. Further, they found an increase in bulk density, attributed to the use of heavy mining machinery, and an alteration of the particle size towards a higher sand and lower silt and clay content on mined fields. The organic carbon content decreased by six to 47%, depending on the age of the mining site, compared to unmined land, and N, P, and K decreased considerably. Similar to our results, a reduction in soil fertility parameters was also observed in open cast coal mining sites. Pandey et al.^27^ found reduced levels of N, P, pH and an increased bulk density at coal mining sites in Eastern India, compared to an unmined reference site. Decreased levels of soil nutrients (N, P, K, iron (Fe), manganese (Mn), copper (Cu), and zinc (Zn)) were found as a result of coal mining in Jharkhand, India^28^.

Farmers’ views about the infrastructural development of the community varied. Some mentioned better access to sand and therefore advantages for construction works, while others highlighted challenges associated with the limited availability of sand and land. This phenomenon has been discussed as a classical “resource curse” for many parts of the Global South^29,30^.

Similarly, the income generated by the youth through sand winning was controversial. While advocates appreciated off-farm job alternatives, especially regarding the decrease in farming opportunities, opponents claimed that such employment was only short-term and would defer the youth from formal education, thereby increasing school-dropout rates. The reduced food availability stated by the farmers corroborate the results of Asare et al.^24^ who found that the average number of meals per day in sand mining communities in Southern Ghana decreased from 3.0 in 2015 to 1.9 in 2020.

Many farmers called for a complete ban on sand mining, however, recent work on sand mining in India shows that banning sand mining without offering alternative supply sources may increase illegality, corruption, and violence in the sector^31^. Therefore, more complex solutions are needed, which involve a more effective collaboration between all stakeholders. This includes sand miners, landlords, farmer representatives, the building industry, regulatory actors, politicians, and city planners. Only concerted action may allow effective zoning of mining areas and farmlands, enforcement to ensure reclamation, and proper compensation payments for farmers^11^. To foster well-informed decisions by policymakers, stakeholders could raise awareness of the effects of sand mining through mass-media such as documentaries^32^. Additionally, the propagation of alternative building materials such as hempcrete, bamboo or cob and recycling of used materials could in the long run help in decreasing the demand for sand^33^.

Our data show that, despite existing legislation, sand miners did not undertake proper reclamation measures and only 3.5% of the fields were levelled. Saviour and Stalin^26^ found that rehabilitated sites had significantly lower yields than unmined sites. They suggest comprehensive rehabilitation measures including the application of P-solubilizing and N-fixing microbes, compost, fertilizer, mulching, planting of selected tree and crop species, and erosion control measures. Limitations of this study include a potential bias in the survey data due to translation problems, as well as a response and memory recall bias due to the respondents’ negative personal experiences with sand mining. Furthermore, the failure of time series remote sensing analyses due to the described constraints highlights the need for further research in this area. To overcome this limitation, future studies could explore alternative methods such as utilising Sentinel 1 active sensors or employing LiDAR drones.

## Conclusions

Our results demonstrate that sand mining on agricultural land greatly affected farming livelihoods and soil fertility parameters in the three sand mining communities around Accra. Structured interviews revealed the long-term loss of agricultural land, the destruction of staple food and cash crops, and the perpetual powerlessness of landless farmers due to mining activities during day and night. While the mining status (mined, near mined or unmined) showed a significant effect on all soil quality parameters except for the C/N ratio, erosion effects (upper and lower slope) only played a minor role.

Further research is needed to assess the locally most cost-effective mitigation strategies against the negative effects of sand mining on agricultural land use. Increased political awareness, stakeholder dialogue, and law enforcement are short-term measures to partially curb the detrimental consequences of sand mining on ecosystem services in Ghana and beyond.

## Data Availability

The datasets used and analysed during the current research are available from the corresponding author upon request.
